# Multi-Element Analysis Based on an Automated On-Line Microcolumn Separation/Preconcentration System Using a Novel Sol-Gel Thiocyanatopropyl-Functionalized Silica Sorbent Prior to ICP-AES for Environmental Water Samples

**DOI:** 10.3390/molecules26154461

**Published:** 2021-07-24

**Authors:** Natalia Manousi, Abuzar Kabir, Kenneth G. Furton, George A. Zachariadis, Aristidis Anthemidis

**Affiliations:** 1 Laboratory of Analytical Chemistry, Department of Chemistry, Aristotle University of Thessaloniki, 54124 Thessaloniki, Greece; nmanousi@chem.auth.gr (N.M.); zacharia@chem.auth.gr (G.A.Z.); 2 International Forensic Research Institute, Department of Chemistry and Biochemistry, Florida International University, Miami, FL 33131, USA; akabir@fiu.edu (A.K.); furtonk@fiu.edu (K.G.F.)

**Keywords:** automation, flow injection, inductively coupled plasma, sol-gel, solid-phase extraction, metals

## Abstract

A sol-gel thiocyanatopropyl-functionalized silica sorbent was synthesized and employed for an automated on-line microcolumn preconcentration platform as a front-end to inductively coupled plasma atomic emission spectroscopy (ICP-AES) for the simultaneous determination of Cd(II), Pb(II), Cu(II), Cr(III), Co(II), Ni(II), Zn(II), Mn(II), Hg(II), and V(II). The developed system is based on an easy-to-repack microcolumn construction integrated into a flow injection manifold coupled directly to ICP-AES’s nebulizer. After on-line extraction/preconcentration of the target analyte onto the surface of the sorbent, successive elution with 1.0 mol L^−1^ HNO_3_ was performed. All main chemical and hydrodynamic factors affecting the effectiveness of the system were thoroughly investigated and optimized. Under optimized experimental conditions, for 60 s preconcentration time, the enhancement factor achieved for the target analytes was between 31 to 53. The limits of detection varied in the range of 0.05 to 0.24 μg L^−1^, while the limits of quantification ranged from 0.17 to 0.79 μg L^−1^. The precision of the method was expressed in terms of relative standard deviation (RSD%) and was less than 7.9%. Furthermore, good method accuracy was observed by analyzing three certified reference materials. The proposed method was also successfully employed for the analysis of environmental water samples.

## 1. Introduction

Natural and anthropogenic processes are two major sources for the continuous release of trace metals in the environment [1]. Some elements or elemental species (e.g., lead, cadmium, mercury, etc.) exhibit toxicity even at low concentration levels [2]. Other metals, such as copper and zinc are essential micronutrients, and they are required for the biological processes of living organisms. As a result, the monitoring of toxic and nutritive elements in environmental samples has aroused considerable concern.

Currently, a wide variety of spectroscopic techniques including flame atomic absorption spectroscopy (FAAS) [3], electrothermal atomic absorption spectroscopy (ETAAS) [4], inductively coupled plasma atomic emission spectroscopy (ICP-AES) [5], and inductively coupled plasma mass spectrometry (ICP-MS) [4] are available for the determination of nutritive and toxic elements [6]. Among the different available spectroscopic techniques, ICP-AES is widely employed in trace and ultra-trace analysis, due to its plethora of benefits, including high sensitivity, extended linear working ranges for the target analytes, as well as its ability to rapidly determine multiple elements [7]. However, the direct determination of elements is a challenging endeavor due to their very low concentration levels, as well as the inherent matrix effects. Therefore, a sample separation/preconcentration step is typically required to improve the sensitivity of common atomic spectroscopic techniques [8]. Flow injection (FI) and related techniques have been proved to be appropriate for on-line fluidic manipulation and for automated sample processing [3]. On-line automated systems are highly attractive sample preparation platforms due to the minimization of reagent consumption and reduced laboratory time and operation cost, in combination with the achievement of high extraction efficiency and enhancement factors [7,9]. 

Solid-phase extraction (SPE) is by far the most attractive approach for sample preparation and preconcentration, since it offers a plethora of benefits including superior performance in terms of straightforward operation, versatility, reliability, and high separation and enrichment capability of the target analytes [10]. Typically, SPE utilizes packed or disk-phase microcolumns, filled with the desired sorbent and placed within the flow network prior to the detection system. As a result, the sorptive phase is considered an integral component of the flow system, and it is repeatedly used for the loading and the elution of the sample solution [9]. However, the applications of on-line automated systems for multi-element separation/preconcentration as a front-end to ICP-AES systems reported in the literature are limited. Chen et al. [11] evaluated the utilization of thiacalix[4]arene tetracarboxylate derivative modified mesoporous TiO_2_ for the extraction of vanadium, copper, lead, and chromium. Peng et al. [12] synthesized a multi-wall carbon nanotube chemically modified silica adsorbent for the on-line SPE of Zn(II), Cu(II), Cd(II), Cr(III), V(V), and As(V) from environmental water samples. Chitosan-based materials have also been reported for the development of on-line platforms for multi-element determination [13,14,15].

Unequivocally, over the past few years, attention has been directed toward the development and evaluation of novel sorptive phases for on-line systems aiming to develop automated methods characterized by high accuracy and sensitivity. In this frame, a variety of sorbents have arisen including carbon nanotubes [16], metal oxides [17], 3D-printed materials [18], and functionalized silicas [19]. Among them, silica-based materials appear to be an excellent choice of support to develop sorptive phases for the extraction of metal ions. Due to their high surface area in combination with the presence of highly reactive silanol groups in its structure, the surface of silicas enables the chemical modification through immobilization of O-, N-, and S-containing organic functional groups [19,20]. Sol-gel technology has been proved to be a significant tool for the preparation of advanced hybrid inorganic–organic polymer coatings. This technology enables the chemical integration of sol-gel sorbent to the substrate in a wide variety of forms (e.g., as particles, fiber, fabric, etc.). Sol-gel materials exhibit various advantages, such as tunable porosity, selectivity, as well as good chemical and thermal stability, and thus, they offer an excellent choice for fabricating automated on-line renewable microcolumn preconcentration platforms for multi-element analysis [3]. Although sol-gel materials have been proved to be powerful sorbents for the microextraction of organic compounds, the applications of sol-gel materials for the development of multi-element analytical techniques are limited, and they are typically applied as in-tube or capillary surface coatings [3,21].

Toward the exploration of sol-gel sorbents for the extraction and preconcentration of metals, Castro et al. [19] prepared a column packed with silica obtained by sol-gel method and functionalized with 2-aminothiazole. The proposed sorbent was employed for the preconcentration of cadmium, copper, and nickel from water samples offering fast kinetics and high adsorption capacity. In 2016, Anthemidis et al. [22] evaluated four different sol-gel sorbents (i.e., sol-gel polytetrahydrofuran, sol-gel polydimethyldiphenylsiloxane, sol-gel triblock copolymers of poly(ethylene oxide) and poly(propylene oxide), and sol-gel graphene) for the flow injection-fabric disk sorptive extraction (FI-FDSE) of lead and cadmium from environmental samples. This approach was an automated alternative to conventional fabric phase extraction (FPSE), which is a novel sample preparation technique introduced by Kabir and Furton [23]. However, the exploration of sol-gel materials in multi-element analytical techniques for the simultaneous determination of toxic and nutrient elements is a new research field that has yet to be explored.

The thiocyanate functional group has long been known for its strong affinity toward metals, and several researchers have reported its use in metal extraction [24,25,26] as well as in creating ion-imprinted polymers for a specific metal ion [27,28]. Although, some of these studies utilized the sol-gel process to implant a thiocyanate functional group on silica substrate, the processes are complex and time consuming and involve multi-steps. To simplify the synthesis process, herein we propose a two-step reaction scheme: (a) acidic hydrolysis of sol-gel precursors, trimethoxysilane and 3-thiocayanatopropyl triethoxysilane; (b) base-catalyzed polycondensation of the hydrolyzed sol-gel precursors that results in a solid three-dimensional network of 3-thiocyanatopropyl functionalized silica. Recently, our research group designed and utilized a 3-thiocyanatopropyl functionalized sol-gel silica sorbent for the automated SPE of Cd(II), Co(II), Cu(II), and Pb(II) coupled with FAAS. This sorbent showed good performance characteristics, and it enabled the sensitive and accurate determination of those analytes in environmental and biological samples. As such, the 3-thiocyanatopropyl-functionalized sol-gel silica sorbent could be a good choice also for multi-elemental extraction/preconcentration of toxic and nutrient metals [29].

In the present study, an automated on-line sample separation and/or preconcentration platform based on the synthesized 3-thiocyanatopropyl-functionalized silica particles was developed as a front-end to inductively coupled plasma atomic emission spectroscopy. The proposed platform was fabricated by packing the sol-gel thiocyanatopropyl-functionalized silica sorbent in an easy-to-repack microcolumn format, and it was coupled with ICP-AES for multi-element analysis. In this way, we aimed to expand the applications of this sorbent and to develop a multi-element method for the simultaneous determination of a wide range of analytes. The chemical and hydrodynamic parameters affecting the performance of the proposed platform were thoroughly investigated and optimized to achieve the highest sensitivity. The accuracy of the proposed method was determined by analyzing spiked samples as well as a certified reference material. Finally, the herein developed platform was successfully employed for the determination of cadmium, lead, copper, chromium, cobalt, nickel, zinc, manganese, mercury, and vanadium in environmental and drinking water samples.

## 2. Results and Discussion

### 2.1. Characterization of the Sol-Gel Thiocyanatopropyl-Functionalized Silica Sorbent

To understand the functional and elemental composition as well as the surface morphology of the particles, thiocyanatopropyl-functionalized sol-gel silica particles were characterized using Fourier transform-infrared spectroscopy (FT-IR) and scanning electron microscopy-energy dispersive spectrometry (SEM-EDS).

#### 2.1.1. Fourier Transform-Infrared Spectroscopy Analysis

**Figure 1:**
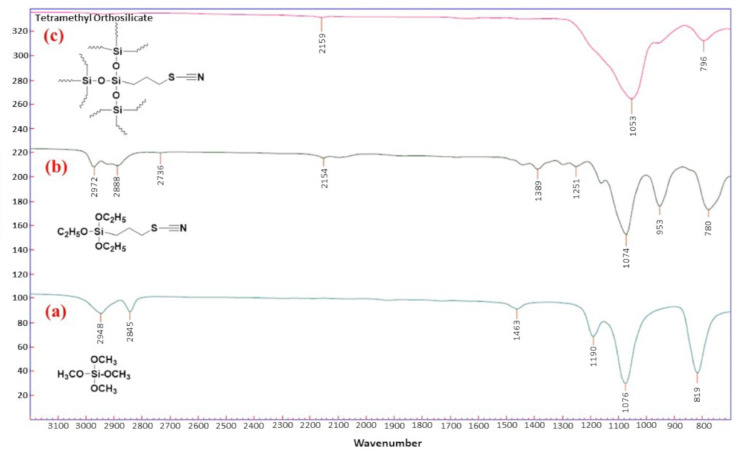
FT-IR spectra of (**a**) tetramethyl orthosilicate (TMOS); (**b**) 3-thiocyanatopropyl triethoxysilane; and (**c**) 3-thiocyanatopropyl-functionalized sol-gel silica sorbent.

The FT-IR spectra of (a) tetramethyl orthosilicate (TMOS), (b) 3-thiocynatopropyl triethoxysilane (3-TCPTES), and (c) 3-thiocyanatopropyl-functionalized sol-gel silica sorbent is depicted in Figure 1. The dominant features of the TMOS FT-IR spectra include bands at 2948 and 2845 cm^−1^ that correspond to the asymmetric and symmetric vibrations of -CH_2_- groups, respectively. The bands at 1463 and 1076 cm^−1^ correspond to the vibration absorption of Si-O-C and Si-O-Si, respectively. The band at 819 cm^−1^ is attributed to Si-C bonds [30]. Major characteristic features of 3-TCPTES FT-IR spectra include bands at 2972, 2157, and 780 cm^−1^, which are attributed to the C-H stretching vibration of CH_2_ group, the C≡N stretching vibration, and the C-S stretching vibration of thiocyanatopropyl functional group. The presence of bands at ~2115, ~1063, and ~796 cm^−1^ in both the 3-TCPTES and 3-thiocyanopropyl-functionalized sol-gel silica FT-IR spectra convincingly suggests the successful integration of 3-thiocyanopropyl functional groups into the 3D sol-gel silica network.

#### 2.1.2. Scanning Electron Microscopy-Energy Dispersive Spectrometry (SEM-EDS) Analysis

The scanning electron microscopy image, presented in Supplementary Materials Figure S1, reveals the rough surface morphology of 3-thiocyanopropyl-functionalized sol-gel silica. As the particles were obtained by manual grinding of a monolithic bed using a mortar and pestle, the particles are not homogeneous in size and shape. A mechanical grinder may provide a monodisperse particle size of the functional silica sorbent. The elemental analysis of sol-gel silica sorbent using EDS revealed the composition as 48.09% Si, 47.13% O, 3.11% S, and 1.67% N. The percentage of S and N in the sorbent can be easily enhanced by adding a higher molar ratio of 3-TCPTES in the sol solution or using only 3-TCPTES as a single sol-gel precursor in the sol solution formulation.

### 2.2. Mechanism of Extraction

**Figure 2:**
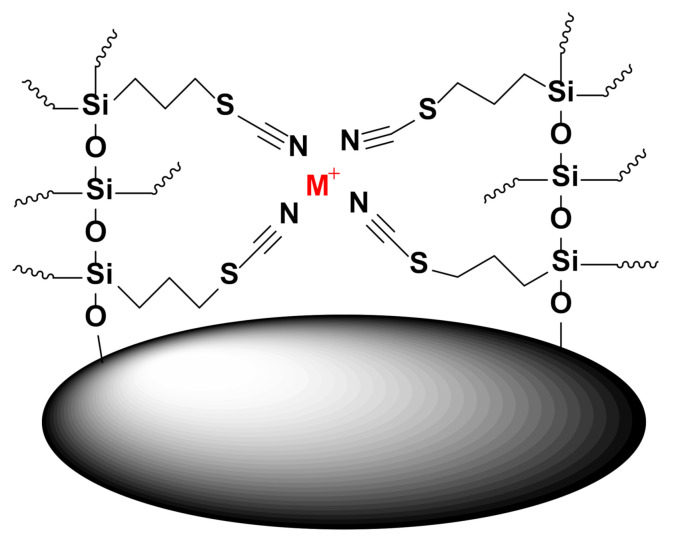
Schematic demonstration of the interaction between 3-thiocyanatopropyl-functionalized sol-gel silica sorbent.

The thiocyanate functional group is known to possess strong affinity toward d-block elements. Schematic representation of the sol-gel sorbent with metal ions (M^+^) was depicted in Figure 2. It can interact with the metal ions either through the nitrogen atom (known as isothiocyanate binding mode) or through the sulfur atom (known as thiocyanate binding mode) [24]. Matveichuk et al. [24] have conducted a detailed study on a group of d-block elements to understand how an individual element interacts with the thiocyanate moiety and experimentally proved that Zn^2+^ and Co^2+^ interact with thiocyanate via nitrogen, whereas Hg^2+^, Mn^2+^, Cd^2+^, Ni^2+^, and Fe^2+^ interact with thiocyanate via the sulfur atom. As such, the thiocyanatopropyl functional group appears to possess a universal affinity toward the d-block elements. When the analytical challenge is to indiscriminately isolate and separate all these elements from the aqueous solution as in the case of wastewater treatment plants, water filtration units, and other water treatment processes, 3-thiocyanaopropyl-functionalized sol-gel silica sorbent may be an ideal choice.

### 2.3. Optimization of the FI-ICP-AES System

The optimization of the ICP-AES parameters is discussed in the Supplementary Materials. For the optimization of the flow injection ICP-AES (FI/ICP-AES) system, the main chemical and hydrodynamic parameters affecting its performance were thoroughly investigated and optimized using the well-established one-variable-at-a-time (OVAT) approach. As such, each parameter was individually examined within a studied range, while all other factors remained constant. For this purpose, a multi-element standard solution containing all studied analytes at a concentration of 25 μg L^−1^ was used throughout the experiments.

The chemical parameters include the pH value of the sample solution, the type, and the concentration of the eluent the eluent and the concentration of the eluent. The pH of the sample is an important factor that significantly affects the performance on-line column preconcentration procedure. A suitable pH value improves the retention efficiency and, in many cases, reduces the matrix interference. The pH value of the sample was investigated in the range of 2.0 to 7.0. Dilute NaOH and HNO_3_ solutions were used to adjust the appropriate pH value. The experimental results are shown in Supplementary Materials, Figure S2 for Cd, Co, Ni, Pb, Zn, Hg and in Figure S3 for Cu, Cr, Mn, and V. As can be observed, the emission intensity was increased by increasing the pH and the extraction efficiency was decreased significant at pH lower than 4 for all analytes. The recorded intensity was the highest from pH 4.0 to pH 6.0 for all studied metal ions. At higher pH, possible hydrolysis phenomena result in gradual signal decrease. As a compromise, a pH value of 5.0 was adopted for further experiments since it was found to be beneficial for most of the target analytes.

The elution procedure of the analytes from the sol-gel thiocyanatopropyl-functionalized silica microcolumn is of a great important to avoid carryover effects that could limit the applicability of the sorbent material. Since the retention efficiency of the analytes was decreased sharply at low pH values, nitric and hydrochloric acid were examined as eluents. However, the utilization of hydrochloric acid did not seem beneficial since it could form salts with some of the target analytes (e.g., mercury and lead), which could sediment in the microcolumn and block the functional groups of the sorbent as well as the frits of the column. Nitric acid solution is an efficient eluent that can elute all retained analytes in a very small segment of the eluent zone. In addition, nitric acid is quite reconcilable with the nebulizer/injector of the ICP atomization system. Diverse nitric acid solutions with concentrations ranged between 0.1 and 2.0 mol L^−1^ were examined. As shown, the intensity increased by increasing the nitric acid concentration up to 1.0 mol L^−1^ and leveled off for concentrations higher than 1.0 mol L^−1^ nitric acid. Thus, 1.0 mol L^−1^ HNO_3_ was used as the eluent during the elution step (Supplementary Materials, Table S2).

A key factor that significantly affects the performance of on-line column preconcentration systems is the loading flow rate (LFR). For a specific time (preconcentration time), LFR determines the volume of sample solution that takes place in the extraction procedure. Although high flow rates are typically desired to achieve high preconcentration factors, adverse phenomena might take place by increasing the flow rate due to column back-pressure, which can negatively impact the sorption efficiency [31]. The effect of LFR on the emission intensity of target ions was studied in the rage 5.0–10.0 mL min^−1^.

As shown in Supplementary Materials, Figure S4, the recorded signal increased practically linearly by increasing the LFR for Cu, Cr, Mn, and V indicating that the adsorption kinetics of the sol-gel thiocyanatopropyl-functionalized silica sorbent is very effective for the studied ions. The same behavior was observed for all analytes. For further experiments, a sample loading flow rate of 10.0 mL min^−1^ was adopted as a compromise considering the sensitivity, the sample consumption, and the time of analysis of the on-line FI/ICP-AES method.

The flow rate (EFR) of the eluent influences the efficiency of the elution procedure of the adsorbed metal ions and the dispersion of the analytes into the eluent zone. Moreover, EFR contributes to the nebulization and atomization process since it defines the amount of analyte mass injected into ICP. The effect of nitric acid flow rate was examined in the range 1.0 to 3.0 mL min^−1^ for an elution time of 30 s (Supplementary Materials, Table S2). The recorded results for all studied analytes show that the signals were increasing up to 2.6 mL min^−1^ and leveled off for higher flow rate. Hence, a flow rate of 2.6 mL min^−1^ was adopted for further experiments.

The on-line flow injection column preconcentration systems are based on the loading time to increase the sensitivity and the enhancement factor (EF). The influence of the loading time (preconcentration time) on the recovery of the metal ions was examined by loading a standard solution containing all target analytes for preconcentration times between 30 to 180 s. The obtained results (Supplementary Materials, Figure S5) revealed a practical, proportionate increase for Cd, Co, Ni, and Zn by increasing the preconcentration time up to 180 s. The same behavior was observed for all analytes. As a compromise between high sensitivity and low time of analysis and sample consumption, a preconcentration time of 60 s was selected. However, it must be noted that if higher sensitivity is required, the preconcentration time can be prolonged based on the needs of the analysis.

### 2.4. Figures of Merit

The analytical performance characteristics of the herein developed method for each metal ion, under the optimized operating conditions and a preconcentration time of 60 s, are presented in Table 1. The sampling frequency of the automatic FI/ICP-AES method was 36 h^−1^. The enhancement factor calculated by the ratio of the slopes of calibration curves with and without preconcentration are given in Table 1.

As can be observed, the enhancement factors ranged between 31 and 53 for the target analytes. The linearity of the proposed method was assessed by linear regression analysis through the construction of calibration curves for multi-element standard solutions subjected to the FI/ICP-AES method. As can be observed, the proposed method exhibited good linearity and a wide linear range for all the examined elements. The detection and quantification limit are calculated by 3 and 10 s criteria, respectively, according to IUPAC recommendation, as 3- or 10-times the standard deviation of the blank solution measurements (*n* = 10) divided by the slope of the corresponding calibration equation. For the target analytes, the LOD values ranged between 0.05 and 0.24 μg L^−1^, while the LOQ values ranged between 0.17 and 0.79 μg L^−1^. The precision of the method was expressed in terms of relative standard deviation (RSD%) for each examined metal at 10.0 μg L^−1^ (*n* = 8) the concentration level varied from 0.8% up to 7.9%.

The accuracy of the developed FI-/ICP-AES multi-element method was examined by analyzing three different standard reference materials: CRM 1643e, IAEA-433, and Seronorm^TM^ Trace Elements Urine Level 1 (4), and it was expressed in terms of relative error between the nominal and experimentally found concentration. As the certified values of cadmium and mercury in Seronorm^TM^ were below the detection limits, they were not evaluated. Student *t*-test was adopted for statistically significant difference between the certified values and the recorded analytical values’ investigation. The calculated *t*_exp_ values are presented in Supplementary Materials, Table S3.

All the *t*_exp_ values are lower than the critical value, *t*_crit, 95%_ = 4.3, meaning that no statistically significant differences were observed for each analyte at a 95% probability level, and the method exhibited satisfactory accuracy.

### 2.5. Interference Studies

The effect of diverse, usually coexisting, ions on the sensitivity of the proposed FI/ICP-AES method for the determination of the studied metal was studied. Consequently, an aqueous solution containing Cd(II), Co(II), Cr(III), Cu(II), Mn(II), Ni(II), Pb(II), Zn(II), Hg(II), and V(II) at a concentration level of 10.0 μg L^−1^ for each metal and the examined interfering ions was analyzed using the developed analytical method. A variant of the emission intensity greater than ±5% was considered as interference. The tolerance limit for the common matrix ions was Na^+^, K^+^ up to 1000 mg L^−1^; Ca^2+^, Mg^2+^ up to 500 mg L^−1^; SO_4_^2−^, NO_3_^−^, HCO_3_^−^ up to 2000 mg L^−1^, and Ag^+^, Al^3+^, Ba^2+^, Fe^3+^ up to 20 mg L^−1^. Hence, the developed method can be used for multi-element analysis in most cases of environmental water samples without applying any masking agent.

### 2.6. Applications in Spiked Environmental Water Samples

The proposed method was applied to the analysis of natural water samples collected from the Northern Greece area: Axios river and Volvi lake. The analytical results are presented in Supplementary Materials, Table S4. The calculated recoveries varied within the range 90.0–106.0%, indicating the applicability of the method for trace multi-element determination in environmental water samples.

### 2.7. Comparison of the Proposed FI/ICP-AES Method with Other Selected On-Line Column Preconcentration ICP-AES Methods

For comparative purposes, the performance characteristics of the proposed method and previously published on-line column preconcentration ICP-AES methods were compared in terms of sampling frequency, detection limit, precision, and enhancement factor, as shown in Table 2.

As can be observed, the precision of the method is similar to or better than the precision of other studies, since similar or better RSD values were obtained. The enhancement factors of the proposed study were better than those reported in most on-line SPE methods [11,12,13,30,31]. However, for Cd and Co [14,15] and Pb [14,15] higher enrichment factors have been reported. Finally, the sensitivity of the proposed method was satisfactory compared to that of the other on-line column preconcentration ICP-AES methods.

The analytical characteristics of the reported study for Cd(II), Co(II), Cu(II), and Pb(II) were also compared to our previously reported study utilizing a 3-thiocyanatopropyl-functionalized sol-gel silica sorbent as a front-end to FAAS [29]. The LODs of this method were lower (i.e., 0.05–0.24 μg L^−1^) compared to the method utilizing FAAS as detection system (i.e., 0.15–1.9 μg L^−1^). On the other hand, higher enrichment factors were obtained for the automated on-line FAAS method (i.e., 73–152), and lower enrichment factors were obtained for the automated on-line ICP-AES method (i.e, 34–53). Moreover, comparable repeatability in terms of RSDs was obtained in both cases for Cd(II), Co(II), and Cu(II) (RSD < 3.9 for the on-line ICP-AES method and RSD < 3.8 for the on-line FAAS method), while the utilization of the on-line FAAs system resulted in better repeatability for Pb(II) (i.e., RSD = 3.8%) compared to that for the on-line ICP-AES system (i.e., RSD = 7.9%). Finally, the utilization of the sol-gel silica sorbent and the ICP-AES detection system enabled the simultaneous determination of Cd(II), Pb(II), Cu(II), Cr(III), Co(II), Ni(II), Zn(II), Mn(II), Hg(II), and V(II) due to the multi-elemental nature of the ICP-AES instrument.

## 3. Materials and Methods

### 3.1. Reagents Materials and Samples

Tetramethyl orthosilicate (TMOS), hydrochloric acid, and isopropanol were purchased from Sigma-Aldrich (St. Louis, MO, USA). 3-Thiocyanatopropyl triethoxysilane was purchased from Gelest Inc. (Morrisville, PA, USA). Ultra-pure deionized water (18.2 MΩ) used in the sol solution synthesis was obtained from a Barnstead NANOPure Diamond (Model D11911) deionized water system (APS Water Services Corporation, Lake Barbara, CA, USA).

Nitric acid (HNO_3_) (65%) and ammonia solution (25%) were supplied by Merck (Darmstadt, Germany). For ultra-pure-quality water, a Milli-Q (Millipore, Bedford, TX, USA) purification system was used during method development, validation, and application. Single stock standard solutions (1000 mg L^−1^) of cadmium, chromium, copper, cobalt, nickel, lead, zinc, manganese, mercury, and vanadium were supplied by Merck (Darmstadt, Germany). The stock standard solutions were prepared in HNO_3_ 0.5 mol L^−1^ for cadmium, chromium, copper, cobalt, nickel, lead, zinc, manganese, and vanadium and in HNO_3_ 2.0 mol L^−1^ for mercury. Multi-element working standard solutions were prepared daily by appropriate serial dilution from the single-element stock standards. The pH of the working solutions was adjusted accordingly to be used in the extraction/preconcentration step.

In order to avoid contamination, laboratory glassware and storage bottles were rinsed with water and soaked in 10% (*v*/*v*) nitric acid overnight. Prior to their use, all sample preparation apparatuses were extensively washed with Milli-Q water.

The following standard reference materials (SRMs) were analyzed for evaluation of the developed method: The NIST (National Institute of Standard and Technology, Gaithersburg, MD, USA) certified reference material (CRM) 1643e, which contains trace elements in water; the International Atomic Energy Agency, IAEA-433, which is a marine sediment; and the Seronorm^TM^ Trace Elements Urine L1. A mass of approximately 0.5 g of sediment SRM was precisely weighed and transferred into Teflon bombs. An appropriate volume of nitric acid perchloric and hydrofluoric acid in a volume ratio of 3/2/1 (HNO_3_/HClO_4_/HF) was added. The certified urine sample was digested using concentrated HNO_3_. The digestion procedure was carried out at 130–140 °C in a shielded Teflon beaker stainless-steel pressurized bomb, according to the manufacturer’s recommendations. After cooling the system, the digests were properly diluted in ultra-pure water and used for the analysis.

Environmental water samples were collected from sampling sites located in Northern Greece during February 2021: Axios river and Volvi lake. All samples were filtered through 0.45 μm membrane filters, acidified to approximately pH 2 with dilute nitric acid and stored at 4 °C in acid-cleaned polyethylene bottles until the analysis. These solutions were used for the determination of the “dissolved” fraction of the metal in the samples.

### 3.2. Apparatus

Centrifugation of different sol solutions to obtain particle free gel was carried out in an Eppendorf Centrifuge Model 5415R (Eppendorf North America Inc., Framingham, MA, USA). A 2510 BRANSON Ultrasonic Cleaner (Branson Inc., Brookfield, CT, USA) was used to obtain bubble-free sol solution prior to the gelation process. An Agilent Carry 670 FT-IR Spectrometer equipped with Universal ATR Sampling Accessory (Agilent Technologies, Santa Clara, CA, USA) was used to perform FT-IR characterization of the sol-gel sorbent. A JEOL JSM 5900 LV scanning electron microscope equipped with an EDS-UTW detector (Jeol USA Inc., Peabody, MA, USA) was used to obtain SEM images as well as elemental composition analysis of the sol-gel sorbent. 

All experiments were carried out using the Optima 3100XL axial viewing inductively coupled plasma atomic emission spectrometer (ICP-AES) of Perkin-Elmer (Norwalk, CT, USA, https://www.perkinelmer.com/) for the multi-element analysis, under the operating conditions presented in Supplementary Materials, Table S1. Optima 3100XL was equipped with an Echelle polychromator (resolution: 0.006 nm at 200 nm) and a segmented-array charge-coupled detector with 235 sub-arrays. Two different spray-chamber/nebulizer configurations, namely, Cyclonic/Babington and Scott double-pass/Gem tip crossflow, were examined considering their efficiency/sensitivity. The wavelengths of the studied elements were fixed according to the sensitivity order of atomic (I) or ionic (II) lines as they are listed in Supplementary Materials, Table S1.

To determine accurate peak wavelengths on peak algorithm, peak area processing was chosen. Due to the transient concentration of analyte into the segment of the eluent, during the elution/measurement step, a read time of 1 s and 10 replicates were adopted for signal recording. The selection of the emission line was based on the sensitivity of the instrument in combination with the absence of spectral interferences.

**Figure 3:**
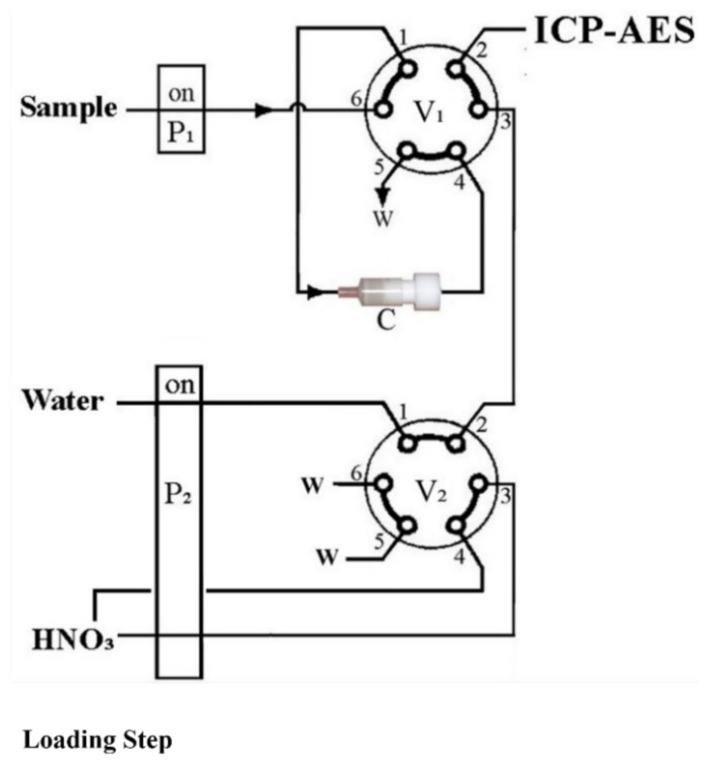
Schematic diagram of the on-line column preconcentration manifold for multi-element analysis by ICP-AES. P_1_, P_2_—peristaltic pumps; V_1_, V_2_—injection valves, V_1_ in “load” position; V_2_ in “A” position; W—waste; C—microcolumn packed with sol-gel thiocyanatopropyl silica sorbent.

The flow injection system for the on-line column preconcentration of the target analytes coupled with the ICP-AES is shown schematically in Figure 3. In brief, it comprises two peristatic pumps (Gilson Minipuls 3, www.gilson.com) for sample (P1) and eluent (P2) solutions delivery, as well as two 6-port 2-position injection valves V_1_ and V_2_ (Supelco Rheodyne, Sigma-Aldrich Chemie GmbH, Taufkirchen, Germany, https://www.sigmaaldrich.com/). The microcolumn C was fixed at ports 1 and 4 of injection valve V1. All tubing of the flow manifold was made of polytetrafluoroethylene (PTFE). The connecting tube between the V_1_ and the nebulizer of ICP-AES was only 50 cm length, 0.5 mm internal diameter to eliminate possible dispersion of the analytes into the segment of the eluent.

A METROHM 654 pH-meter (Metrohm AG, Herisau, Switzerland) was employed for the pH adjusting of sample solutions.

### 3.3. Synthesis of 3-Thiocyanatopropyl-Functionalized Sol-Gel Silica Sorbent

The sol solution was prepared by sequential addition of tetramethyl orthosilicate (TMOS), 3-thiocyanatopropyl triethoxysilane, and 2-propanol in a 50 mL amber reaction bottle at a molar ratio of 1:0.3:15, respectively, with vortexing for 2 min after adding each ingredient. Subsequently, 0.1 mol L^−1^ HCl was added to the mixture at a molar ratio between TMOS and 0.1 mol L^−1^ HCl 1:5. After thorough mixing of the sol solution, the mixture was subjected to prolonged hydrolysis at 50 °C for 8 h. The solution was then transferred into a wide-mouth glass reaction vessel and a Teflon-coated bar magnet was added to it. In order to initiate polycondensation, 1.0 mol L^−1^ NH_4_OH (impregnated with 0.25 mol L^−1^ NH_4_F) was added in droplets under constant stirring on a magnetic stir bar. The ratio between TMOS and 1.0 M NH_4_OH was maintained at 1:1.2. The sol solution was turned into transparent gel in 30 min. The sol-gel monolithic bed was thermally conditioned and aged for 24 h at 50 °C.

Subsequently, the monolithic bed was crushed and subjected to drying for 24 h at 70 °C. The dried sol-gel sorbent was then crushed and pulverized into fine particles using a mortar and a pestle. To clean the sol-gel sorbent from unreacted precursors, solvent, and reaction by-products, the powder was loaded in a fiber-glass thimble and subjected to Soxhlet extraction for 4 h using methanol:methylene chloride 50:50 (*v*/*v*) as the cleaning solution. The sol-gel sorbent was then dried at 70 °C for 24 h. The dried sol-gel sorbent was then ready for upstream processing such as characterization and application. The construction of the repacked microcolumn is described in the Supplementary Μaterials.

### 3.4. Automatic On-Line Operational Procedure

The automatic on-line flow injection microcolumn preconcentration analytical procedure for the multi-element determination was operated in four main steps, which are presented in Supplementary Materials, Table S2.

In case a new sample or standard solution was introduced for the first time, a pre-fill step was utilized to fill the tubing of the pump P_1_ up to valve V_1_.

In loading steps (step 1 and 2, Figure 1), valve V1 was switched to the “load” position, while P_1_ was turned on for sample or standard solution delivery through the microcolumn at flow rate of 6.4 mL L^−1^. The analytes were quantitatively retained in the microcolumn. For the elution (step 3), the injection valve V_1_ was in the “elute” position. The eluent 1.0 mol L^−1^ HNO_3_ was delivered through the microcolumn to desorb and to deliver the analytes into the ICP nebulizer and plasma torch for atomization.

This step lasts 30 s, which was found to be appropriate for complete washing of the column. It is noteworthy that the eluent flows through the microcolumn in the opposite direction to that of the sample or standard solution, resulting in minimum dispersion of the analytes into the eluent segment. In this way, gradual one-side compaction of the sorbent was also evaded. In all cases, five replicate measurements were performed.

## 4. Conclusions

In this study, a streamlined approach for synthesizing thiocyanatopropyl-functionalized silica sorbent using sol-gel process was described. The sol-gel based sorbent is deployed in an on-line automated sample preconcentration and separation platform utilizing an easy-to-repack microcolumn for ICP-AES multi-elemental analysis. The novel type of constructed microcolumn reveals some extra advantages such as convenience in preparation and handling on a flow injection manifold. The packing of the column with the sol-gel thiocyanatopropyl-functionalized silica offered very low flow resistance, excellent packing reproducibility, good preconcentration efficiency, as well as satisfactory sensitivity and high reusability calculated as more than 700 loading/elution cycles. It can be concluded that the sol-gel chemistry is an interesting alternative to the development of metal adsorbents, without requiring the addition of chelating agents for complex formation. The proposed method is fast, simple, sensitive, and selective for the multi-element determination of metals in environmental water and urine samples.

## Figures and Tables

**Table 1:** Figures of merit of the proposed FI/ICP-AES method (for 60 s preconcentration time).

	Cd(II)	Co(II)	Cr(III)	Cu(II)	Mn(II)	Ni(II)	Pb(II)	Zn(II)	Hg(II)	V(II)
Enhancement factor	53	35	46	34	36	36	46	34	39	31
Linear range (μg L^−1^)	0.33–100	0.17–80	0.44–80	0.20–100	0.26–50	0.49–80	0.79–100	0.33–80	0.62–80	0.18–80
Correlation coefficient (*r*)	0.9993	0.9981	0.9954	0.9978	0.9984	0.9993	0.9985	0.9988	0.9986	0.9992
Sensitivity (slope), μg^−1^ L	169.0	197.6	3323.9	5206.1	4072.5	38.8	11.4	160.1	199.7	911.3
Detection limit (3 s), μg L^−1^	0.10	0.05	0.13	0.06	0.08	0.15	0.24	0.10	0.18	0.05
Quantification limit (10 s), μg L^−1^	0.33	0.17	0.44	0.20	0.26	0.49	0.79	0.33	0.62	0.18
Precision (RSD, *n* = 8), %	3.9	2.6	0.8	1.6	2.0	2.2	7.9	2.5	1.7	2.9
Slope (without preconcentration), μg L^−1^	3.18	5.68	72.33	155.38	112.93	1.07	0.25	4.76	5.16	29.22

**Table 2:** Comparison of the performance characteristics of the developed method against selected on-line SPE procedures for multi-element determination with ICP-AES.

Sorbent	Analytical Characteristics	Cd	Co	Cr	Cu	Hg	Mn	Ni	Pb	Zn	V	Ref.
Modified mesoporous TiO_2_	*c*_L_ *s*_r_ (%) PF	-	-	0.15 2.9 20	0.23 3.9 20	-	-	-	0.50 4.6 20	-	0.09 1.7 20	[11]
MWCNTs chemically modified silica	*c*_L_ *s*_r_ (%) PF	0.11 3.1 10	-	0.27 3.1 10	0.91 4.0 10	-	-	-	-	0.45 4.1 10	0.55 7.3 10	[12]
Chitosan-modified ordered mesoporous silica	*c*_L_ *s*_r_ (%) PF	0.05 4.0 20	-	-	0.30 6.7 20	0.93 5.3 20	-	-	0.96 1.8 20	-	0.33 2.8 20	[13]
N-(2-hydroxyethyl) glycine-type chitosan chelating resin	*c*_L_ *s*_r_ (%) EF	0.004 NA 106	0.023 NA 87	-	0.068 NA 32	-	0.018 NA 14	0.13 NA 16	0.085 NA 25	0.05 NA 21	0.17 NA 18	[14]
EDTriA-type chitosan chelating resin	*c*_L_ *s*_r_ (%) EF	0.002 <10 116	0.022 <10 93	-	0.066 <10 35	-	0.018 <10 14	0.12 <10 16	0.080 <10 112	0.048 <10 25	0.15 <10 19	[15]
HyperSep SCX	*c*_L_ *s*_r_ (%) EF	0.07 4.5 18.4	0.06 3.7 18.4	0.2 3.9 18.4	0.08 4.5 18.4	-	0.1 3.7 18.4	0.07 4.2 18.4	0.2 2.5 18.4	0.08 4.2 18.4	-	[31]
AG50W-X8 Cation exchange	*c*_L_ *s*_r_ (%) PF	1.0 2.5 10	-	-	-	-	-	4.0 2.6 10	2.0 4.0 10	-	-	[32]
Sol-gel-functionalized silica	*c*_L_ *s*_r_ (%) EF	0.10 3.9 53	0.05 2.6 35	0.13 0.8 46	0.06 1.6 34	0.18 1.7 39	0.08 2.0 36	0.15 2.2 36	0.24 7.9 46	0.10 2.5 34	0.05 2.9 31	This work

*f*, sampling frequency; *c*_L_, detection limit (values in μg L^−1^); *s*_r_, relative standard deviation; EF, enhancement or preconcentration factor based on their availability, NA, not available.

## Data Availability

Not applicable.

## Supplementary Materials

The following are available online. Table S1. Instrumental conditions and description of the ICP-AES system. Table S2. Operation sequences of the on-line microcolumn preconcentration system coupled with ICP-AES, Table S3. Determination of trace metals in certified reference materials with the proposed FI/SPE-ICPAES method. Mean value ± standard deviation based on three replicates (*n* = 3), t_exp_._,_ experimental value; t_crit_. = 4.3 (at 95 % probability level). Table S4. Multi-element analysis of trace metals in spiked natural water samples. Figure S1. SEM-EDS image of 3-thiocyanatopropyl-functionalized sol-gel silica particles (at 5.00× magnification). Figure S2. Effect of pH on the emission intensity of Cd, Co, Ni, Zn, Pb, and Hg at the 25 μg L^−1^ concentration level of each metal ion. All other experimental parameters as presented in Table S1. Figure S3. Effect of pH on the emission intensity of Cu, Cr, Mn, and V at the 25 μg L^−1^ concentration level of each metal ion. Figure S4. Effect of loading flow rate on the emission intensity of Cu, Cr, Mn, and V at 25 μg L^−1^ concentration level each metal ion. All other experimental parameters as presented in Table S1. Figure S5. Effect of preconcentration time on the emission intensity for Cd, Co, Ni, and Zn at the 10.0 μg L^−1^ concentration level of each metal ion. All other experimental parameters as presented in Table S1. Figure S6. Construction and the main components of the repacked microcolumn. All other experimental parameters as presented in Table 1. Construction of the repacked microcolumn, examination of the ICP-AES parameters, interference studies.

## Sample Availability

Samples of the compounds are not available from the authors.
