# Supplementary material for: Multi-Element Analysis Based on an Automated On-Line Microcolumn Separation/Preconcentration System Using a Novel Sol-Gel Thiocyanatopropyl-Functionalized Silica Sorbent Prior to ICP-AES for Environmental Water Samples
Source: Molecules. 2021 Jul 24;26(15):4461. doi: 10.3390/molecules26154461 (PMC8347399; doi:10.3390/molecules26154461)
Supplement: Supplementary file 1 [file molecules-26-04461-s001.zip › molecules-1309222-supplementary.pdf]

molecules

SUPPLEMENTARY MATERIAL

Multi-element Analysis Based on an Automated On-Line Microcolumn Separation/Preconcentration System Using a Novel Sol-Gel Thiocyanatopropyl-Functionalized Silica Sorbent Prior to ICP-AES for Environmental Water Samples

Natalia Manousi [1], Abuzar Kabir [2], Kenneth G. Furton [2], George A. Zachariadis [1] and Aristidis Anthemidis [1,*]

[1] Laboratory of Analytical Chemistry, Department of Chemistry, Aristotle University of Thessaloniki, 54124 Thessaloniki, Greece; nmanousi@chem.auth.gr (N.M.); zacharia@chem.auth.gr (G.A.Z.)
[2] International Forensic Research Institute, Department of Chemistry and Biochemistry, Florida International University, Miami, FL 33131, USA; akabir@fiu.edu (A.K.); furtonk@fiu.edu (K.G.F.)
* Correspondence: anthemid@chem.auth.gr

Table S1. Instrumental conditions and description of the ICP-AES system

Parameter	*Value / Description*
RF generator	40 MHz, free-running
RF Incident Power	1400 W (optimized)
Viewing mode	Axial
Torch	Fassel type
Injector, i.d.	Quartz glass, 2.0 mm
Spray Chamber	Cyclonic type (optimized)
Nebulizer	Babington type (optimized)
Auxiliary Ar gas flow rate	0.6 L min^{-1}
Plasma gas Ar flow rate	15.0 L min^{-1}
Nebulizer Ar gas flow rate	0.85 L min^{-1}
Waste uptake flow rate	3.5 mL min^{-1}
Target analytes	*Spectral lines (nm)*
Cd	214.440(II) / 228.802(I)
Co	228.616(II) / 238.892(II)
Cr	283.563(II) / 357.869(I)
Cu	324.752(I) / 224.700(II)
Hg	253.652(I) / 302.150(I)
Mn	257.610(II) / 259.372(II)
Ni	232.003(II) / 221.648(II)
Pb	220.353(II) / 217.000(I)
Zn	213.857(I) / 202.548(II)
V	292.402(II) / 309.310(II)

Table S2. Operation sequences of the on-line microcolumn preconcentration system coupled with ICP-AES

Step	V_1	V_2	P1	P2	Delivered medium	Flow rate (mL min^{-1})	Time (s)	Operation
Pre-fill	Elute	A	on	on	Sample or standard solution	10.0	10	Prefilling the tubes
1	Load	A	on	on	Sample or standard solution / Water	10.0	50	Preconcentration
2	Load	B	on	on	Sample or standard solution / 1.0 mol L^{-1} HNO_3	10.0 & 2.6	10	Preconcentration / Plasma equilibration with eluent flow
3	Elute	B	off	on	1.0 mol L^{-1} HNO_3	2.6	30	Elution / Measurement
4	Elute	A	off	on	1.0 mol L^{-1} HNO_3	2.6	10	Neutralization of microcolumn

Table S3. Determination of trace metals in certified reference materials with the proposed FI/SPE-ICPAES method. Mean value ± standard deviation based on three replicates (n = 3), $t_{exp.}$, Experimental value, $t_{crit.}$ = 4.3 (at 95 % probability level).

Certified Reference Material	Cd	Co	Cr	Cu	Hg	Mn	Ni	Pb	Zn	V
**CRM 1643e (Trace Element Water)**										
Certified value (μg L^{-1})	6.568 ± 0.073	27.06 ± 0.32	20.4 ± 0.24	22.76 ± 0.31	-	38.97 ± 0.45	62.41 ± 0.69	19.63 ± 0.21	78.5 ± 2.2	37.86 ± 0.59
Found* (μg L^{-1})	6.2 ± 0.3	26.3 ± 1.2	19.1 ± 1.4	21.9 ± 1.2	-	40.2 ± 2.1	64.7 ± 3.9	18.5 ± 1.3	80.3 ± 4.6	36.2 ± 2.4
Relative Error	- 5.6	- 2.8	- 6.4	- 3.8	-	3.2	3.7	- 5.8	2.3	-4.4
$t_{exp.}$***	1.99	1.10	1.61	1.24	-	-1.01	-1.02	1.51	-0.68	1.20
**IAEA-433 (Marine sediment)**										
Certified value (mg kg^{-1})	0.153 ± 0.033	12.9 ± 1.2	136 ± 10	30.8 ± 2.6	0.338 ± 0.017	316 ± 16	39.4 ± 3.1	26.0 ± 2.7	101 ± 8	160 ± 11
Found* (mg kg^{-1})	0.15 ± 0.01	12.4 ± 0.6	134 ± 7	32.0 ± 1.9	0.31 ± 0.03	304 ± 12	37.8 ± 2.4	27.1 ± 1.7	99 ± 3.1	151 ± 11
Relative Error	- 5.2	-3.9	- 1.5	3.9	- 9.8	- 3.8	- 4.1	4.2	- 2.0	- 5.6
$t_{exp.}$	1.98	1.44	0.50	-1.09	2.20	1.73	1.16	-1.12	1.12	1.42
**Seronorm™ (Trace Element Urine)**										
Certified value (mg kg^{-1})	0.20 ± 0.04	0.72 ± 0.15	0.54 ± 0.12	31 ± NP	0.036 ± 0.014	0.73 ± 0.15	1.51 ± 0.3	0.68 ± 0.13	334 ± 67	0.66 ± 0.08

Found* (mg kg^{-1})	<LOD	0.68 ± 0.06	0.50 ± 0.04	31.5 ± 1.5	<LOD	0.75 ± 0.03	1.45 ± 0.09	<LOD	320 ± 15	0.61 ± 0.05
Relative Error	-	- 5.6	- 7.4	1.6	-	2.7	-4.0	-	- 4.2	-7.6
$t_{exp.}$	-	1.12	1.73	-0.58	-	-1.16	1.16	-	1.62	1.73

NP: Not provided.

Table S4. Multielement analysis of trace metals in spiked natural water samples.

**Sample**	**Cd(II)**	**Co(II)**	**Cr(III)**	**Cu(II)**	**Hg(II)**	**Mn(II)**	**Ni(II)**	**Pb(II)**	**Zn(II)**	**V(II)**
**River water**	< LOD	1.9 ± 0.1	< LOD	4.8 ± 0.4	< LOD	6.2 ± 0.3	3.8 ± 0.2	< LOD	12.3 ± 0.9	7.4 ± 0.4
Added	5.0	5.0	5.0	5.0	5.0	5.0	5.0	5.0	5.0	5.0
Found	4.8 ± 0.2	6.5 ± 0.3	4.5 ± 0.3	9.7 ± 0.6	4.6 ± 0.4	11.1 ± 0.8	8.7 ± 0.5	5.3 ± 0.5	17.2 ± 0.8	12.1 ± 0.8
Recovery (%)	96	92	90	98	92	98	98	106	98	94
**Lake water**	< LOD	< LOD	1.3 ± 0.1	3.5 ± 0.3	< LOD	4.6 ± 0.3	5.5 ± 0.4	2.8 ± 0.3	9.5 ± 0.5	3.3 ± 0.2
Added	5.0	5.0	5.0	5.0	5.0	5.0	5.0	5.0	5.0	5.0
Found	4.7 ± 0.3	4.9 ± 0.2	6.5 ± 0.2	8.6 ± 0.5	4.7 ± 0.3	9.5 ± 0.5	10.4 ± 0.4	7.6 ± 0.6	14.3 ± 0.9	8.2 ± 0.4
Recovery (%)	94	98	104	102	94	98	98	96	96	98

[a] Concentrations in μg L^{-1}, mean value ± standard deviation; LOD: Limit of detection.

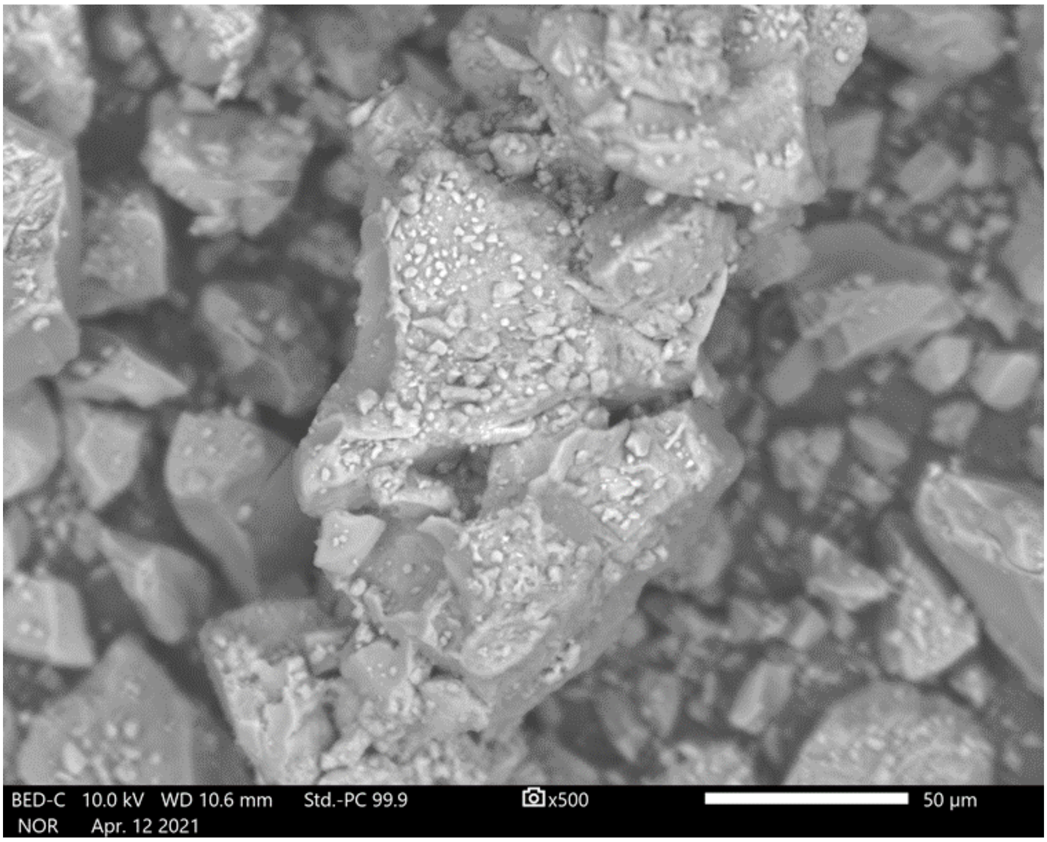

Figure S1. SEM-EDS image of 3-thiocyanatopropyl functionalized sol-gel silica particles (at 5.00× magnification).

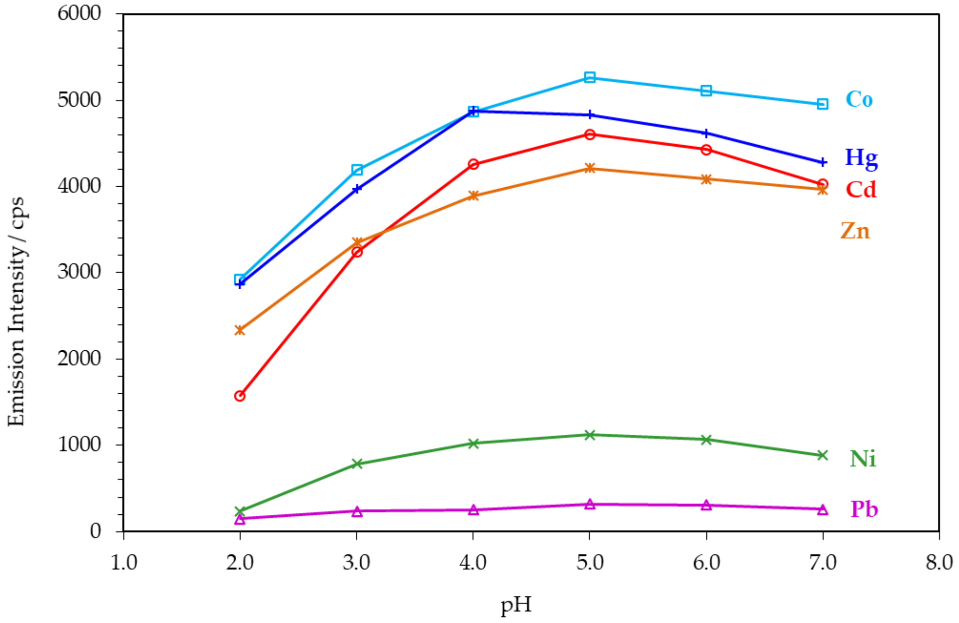

Figure S2. Effect of pH on the emission intensity of Cd, Co, Ni, Zn, Pb and Hg at 25 μg L^{-1} concentration level each metal ion. All other experimental parameters as presented in Table S1.

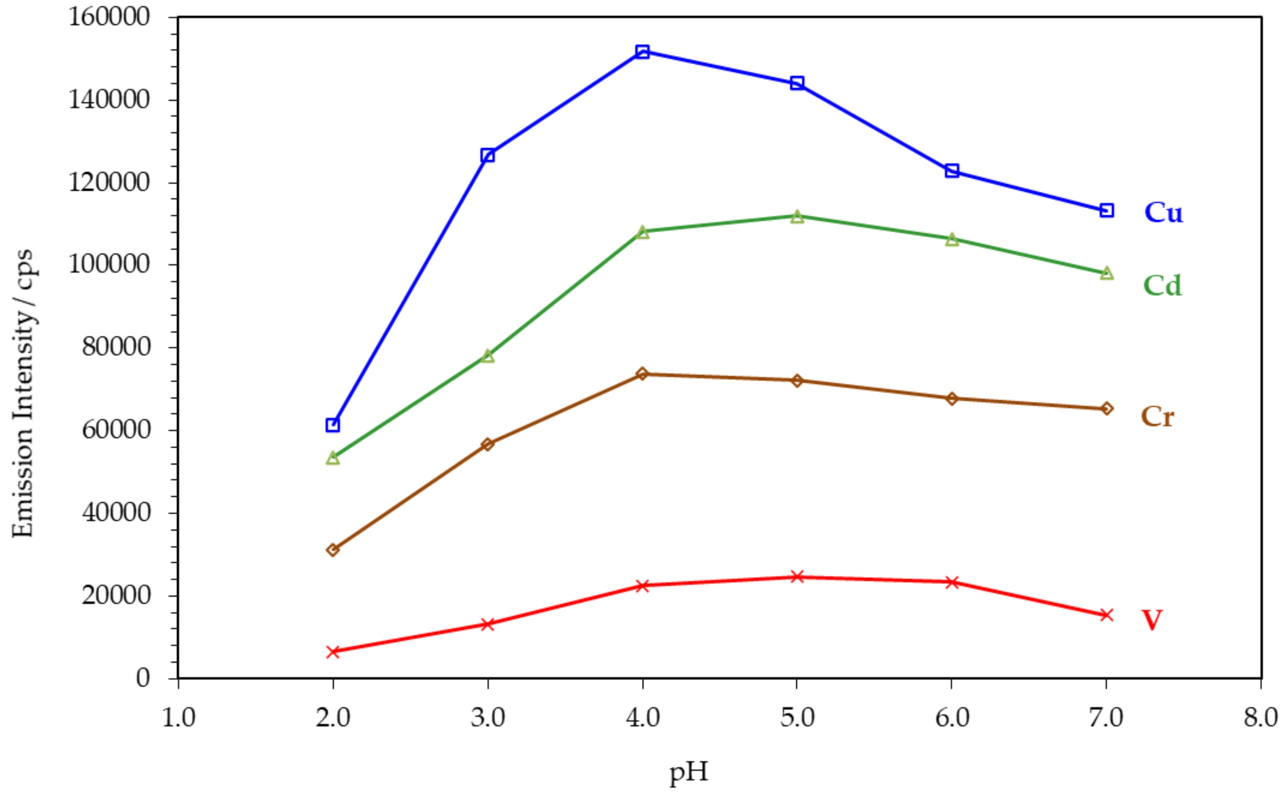

Fig. S3. Effect of pH on the emission intensity of Cu, Cr, Mn and V at 25 μg L^{-1} concentration level each metal ion. All other experimental parameters as presented in Table 1.

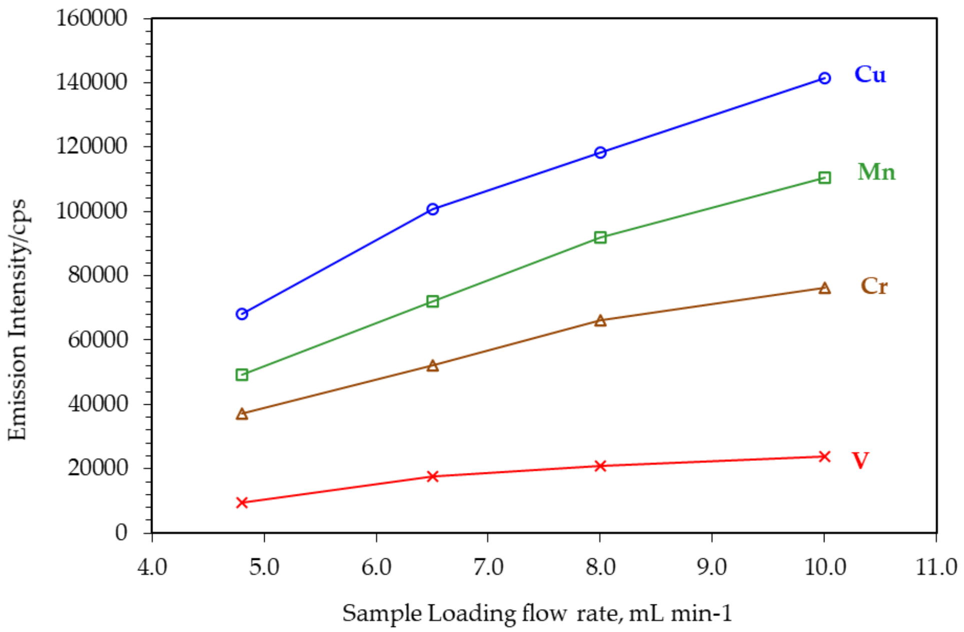

Figure S4. Effect of loading flow rate on the emission intensity of Cu, Cr, Mn and V at 25 µg L^{-1} concentration level each metal ion. All other experimental parameters as presented in Table S1.

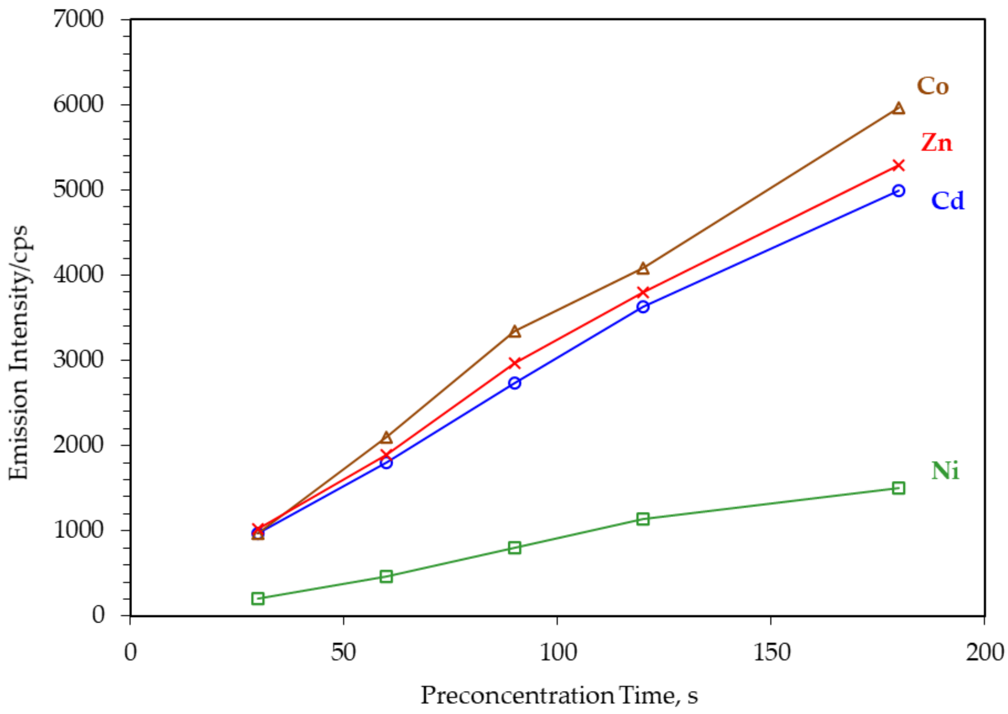

Figure S5. Effect of preconcentration time on the emission intensity for Cd, Co, Ni and Zn at 10.0 μg L^{-1} concentration level each metal ion. All other experimental parameters as presented in Table S1.

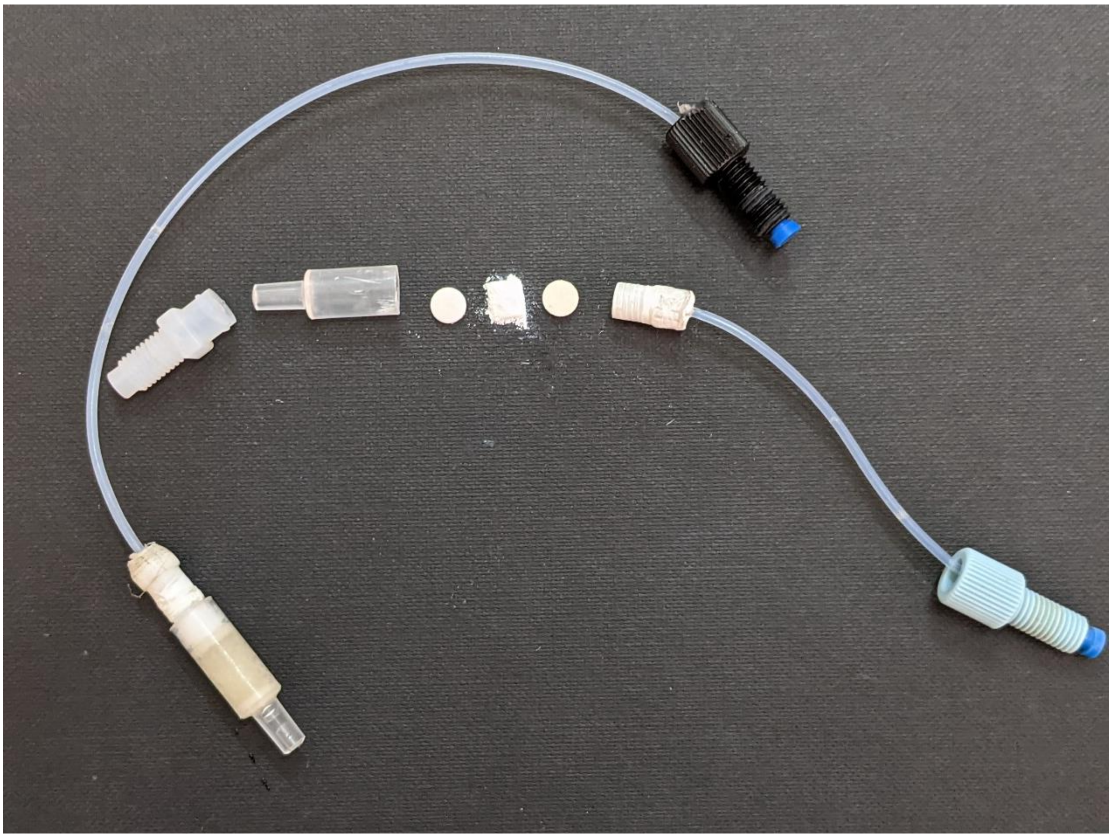

Figure S6. Construction and the main components of the repacked microcolumn

Construction of the repacked Microcolumn

The microcolumn was constructed from a polypropylene 1.0 mL disposable syringe (length 10 cm; i.d. 5.0 mm) with luer slip centered tip. The main body of syringe was cut properly at a length ca. 15.0 mm, thus the resulted repacked-microcolumn had an effective length varied from 5.0 to 10.0 mm and an internal diameter of 5.0 mm offering limited backpressure. An amount of 120 mg of sol-gel thiocyanatopropyl functionalized silica sorbent was firmly packed into the column and blocked by commercial frits at both ends for the sorbent immobilization, as it is shown in Figure S6. Push-fit connections were used to install the microcolumn in the FI manifold, at ports 1 and 4 of the injection valve V_1 (Figure 3). This configuration facilitated rapid replacement and re-packing of the micro-column, whenever required, thereby overcoming the deterioration of the analytical performance of the method due to the progressive deactivation of the sorbent material. Prior to the utilization of a fresh column, the sol-gel thiocyanatopropyl silica sorbent was flushed with 1.0 mol L^{-1} HNO_3 and deionized water. The arrangement of the microcolumn ensured the simple and stable flow of the sample solution and the eluent through the sorbent. As a result, effective contact between the novel sol gel sorbent and the target analytes was achieved. The microcolumn packed with sol-gel thiocyanatopropyl silica sorbent, was proven to operate reliably for at least 700 sorption/elution cycles.

Examination of the ICP-AES parameters

Between the two most sensitive emission spectral lines (Table S1), these with the higher slope of the calibration curves, for each analyte, are adopted for further study namely: Cd 228.802 nm; Co 238.892 nm; Cr 357.869 nm; Cu 324.752 nm; Hg 302.150 nm; Mn 257.610 nm; Ni 232.003 nm; Pb 220.353 nm; Zn 213.857 nm; V 309.310 nm.

Two different configurations of nebulization systems were examined by means of increased sensitivity: Babington-type nebulizer in a cyclonic spray chamber and a crossflow nebulizer in a Scott double-pass spray chamber. Higher emission intensity were reported with Babington / Cyclonic configuration coupled with the proposed on-line microcolumn preconcentration system for all analytes. This combination exhibits higher analyte mass transportation into the plasma because cyclonic spray chamber can effectively manipulate higher amounts of sample solution.

The influence of radio frequency (RF) power on the intensity was studied between 1200-1400 W for each element. It is known that the RF power affects positively the plasma temperature. Thus, an increase of RF power can result in higher intensities for the recorded emission lines. As a result, intensity increased by increasing the RF power up to 1400 W for all studied elements. On the contrary, plasma extinction occurred for RF values lower than 1200 W. Thus, further experiments were conducted at a RF incident power of 1400 W. Subsequently, the effect of the nebulizer gas flow rate on the emission intensity was investigated in the range 0.6-1.0 L min^{-1}. It was observed that an increase of the flow rate up to 0.85 L min^{-1} enhanced the recorded emission intensity for all analytes. Thus, nebulizer gas flow rate of 0.85 L min^{-1} was chosen for the experimental procedure.
